# Characterization of Residual Stresses in Composite Parts Manufactured by Material Extrusion Technology Using Reflection Photoelasticity

**DOI:** 10.3390/polym18040442

**Published:** 2026-02-10

**Authors:** Karol Goryl, Marek Kočiško, Radoslav Vandžura, Peter Frankovský

**Affiliations:** 1 Faculty of Manufacturing Technologies, Technical University of Kosice, Bayerova 1, 080 01 Presov, Slovakia; karol.goryl@tuke.sk (K.G.); radoslav.vandzura@tuke.sk (R.V.); 2 Faculty of Mechanical Engineering, Technical University of Kosice Kice, Letná 9, 042 00 Kosice, Slovakia; peter.frankovsky@tuke.sk

**Keywords:** residual stress, reflection photoelasticity, material extrusion, abrasive water jet

## Abstract

Residual stresses are a persistent challenge in the additive manufacturing of composite parts by FFF (Fused Filament Fabrication) and can impair dimensional accuracy and mechanical performance. This article evaluates reflection photoelasticity (PhotoStress) as a full-field optical technique to visualize and compare residual-stress relaxation in ASA (Acrylonitrile Styrene Acrylate) reinforced with aramid fibers. The approach combines a controlled AWJ (Abrasive Water Jet) relief cut to induce local stress release with subsequent optical recording of isochromatic fringe fields using a reflection polariscope. Samples with thicknesses of 2–10 mm were manufactured and evaluated in two conditions: non-annealed and after annealing (80 °C/5 h). Under identical optical settings, no discernible isochromatic fringes were detected for 2–6 mm (Nmaxlobal < 0.60 in both conditions), whereas resolvable fringe patterns were observed for 8–10 mm. For 8 mm, the response was localized near the relief cut, with Nmax,global = 1.0 in the non-annealed condition and Nmax,global < 0.60 after annealing. For 10 mm, the response was more spatially extensive, and annealing reduced the global maximum from Nmax,global = 1.2 to 0.9. Taken together, these results demonstrate that reflection photoelasticity supports comparative full-field visualization of residual-stress relaxation in FFF composite specimens under fixed measurement conditions. In addition, an AWJ relief cut constitutes a practical and repeatable stress-release feature with limited additional thermal influence in the present configuration.

## 1. Introduction

Currently, additive manufacturing methods for components focus on the possibilities of using new materials, namely composites. The advantage of composite components produced by additive technologies is their higher structural strength, resistance to chemicals, and, in certain cases, reduced weight. In the technology of material extrusion, there are several composite printing materials, where the primary concern is adding chopped fibers to the basic thermoplastic material. The most used fibers include carbon, aramid, glass, and wood. Printing such materials requires careful adjustment of the printing process parameters so that the desired quality and functionality of the components can be achieved [1,2,3].

For this reason, a large amount of research and experiments are devoted to the correct setting of printing process parameters (nozzle temperature, heating plate temperature, printing speed, heating chamber temperature). Incorrectly set process parameters can lead to unpredictable errors during printing, which subsequently affect the integrity of the printed component. Basic errors include high porosity between layers of material, peeling of the first layers from the printing plate, or the formation of residual stresses that lead to other deformations [1,4]. From the perspective of additive technology in composite extrusion printing, several design solutions for composite printing are distinguished:

- Printing with two nozzles: This is a process of selective 3D (three-dimensional) printing using two materials, i.e., filaments, where printing works either with two nozzles or with a more advanced filament exchange system. The method works on the principle of alternating application of materials in one printing process. The advantage of this method is the possibility of checking mechanical properties already at the design stage, because it is possible to design and create various composite structures [5].
- Coating fibers in the nozzle: To print composite components in this way, it is necessary to provide two sources of materials, one for the polymer matrix (filament) and the other for the reinforcement (fiber). This method is based on the principle of mixing continuous reinforcing fiber with a thermoplastic material. During printing, the thermoplastic material is fed into the nozzle together with the reinforcing fiber, where the thermoplastic material coats the reinforcing fiber when heated and is then extruded onto the print bed [6,7].
- Printing with CFF (Continuous Filament Fabrication) technology: CFF technology is patented by Markforged and represents a new way of 3D printing composites. The printer works on the FFF principle, with the difference that the print head consists of two nozzles. The principle of 3D printing is as follows: through the first nozzle, a thermoplastic material is printed, which forms a matrix, and through the second nozzle, reinforcement in the form of continuous fibers is deposited into the molten material. The advantage of this technology is the use of continuous fibers, which can better absorb and distribute shocks and at the same time approach the strength of metal [8].

Residual stresses also occur in composite materials, since during the production of composite components, hardening or melting occurs at high temperatures. The presence of high temperatures results in the formation of residual stresses in the composite material. The main causes of the formation of such stresses in composite materials are the different mechanical properties of the matrix and the reinforcement used, and finally, temperature fluctuations and gradients during the extrusion process. Other sources of stress formation can also include humidity and differences in the relative volume of fibers in the matrix [9].

In the case of additive manufacturing, residual stresses have a negative impact on the quality and performance of the printed component. The impact and characteristics of residual stress depend on the additive manufacturing technology, materials, printing input parameters, and external influences. In metal printing technology, residual stresses significantly reduce the lifespan of the printed component, because they act on the given component as a force that causes cracks and other deformations during cyclic loading. During the printing process, residual stresses affect the dimensional accuracy of the component, cause delamination of layers at the edges, and, at the same time, reduce static and dynamic resistance [9].

## 2. State of the Art

Most of the research and experiments conducted deal with the relationship between the settings of printing process parameters and the formation of residual stresses. From the overview, it can be concluded that a high level of research is focused on additive manufacturing technologies, FFF, SLA (Stereolithography), and PBF (Powder Bed Fusion). The trend today is the evaluation and quantification of residual stresses in additive manufacturing technology FFF, thanks to its wide use in industry and the availability of materials.

Hussein Alzyod and Peter Ficzere investigated residual stresses and warpage in FFF technology. They investigated the influence of common printing parameters (printing temperature, printing speed, bed temperature, infill density, layer thickness, and infill pattern) on residual stress and warpage for PEI (Polyetherimide), ABS (Acrylonitrile Butadiene Styrene), and PA6 (Polyamide 6) materials. They used numerical simulations in Digimat-AM in combination with Taguchi’s orthogonal array L27 and ANOVA (Analysis of Variance) analysis, performing 81 simulation runs. They found a strong material dependence of the dominant parameters: for PEI and ABS, layer thickness was the most critical, while for PA6, printing temperature dominated. The infill pattern consistently contributed significantly to reducing warpage in all three materials, supporting the need for material-specific optimization of printing conditions [10].

Hamed Bakhtiari et al. reviewed the impact of printing parameters on the fatigue properties of FFF-produced parts. The aim of the work was to synthesize knowledge on how printing orientation, infill density and pattern, layer height, and nozzle temperature affect fatigue life in tension, compression, and bending. The authors used a systematic literature review and considered relevant standards for polymer fatigue testing (e.g., ASTM D7774, ASTM D7791) and their applicability to anisotropic FFF materials. The summarized results indicated that higher infill density and lower layer height generally improve fatigue resistance, while the impact of other parameters (e.g., raster angle) is significantly dependent on the type of loading. They also stated that the so-called “on edge” orientation (in the Y axis) often leads to better fatigue performance compared to the X and Z orientations, supporting the need for targeted parameter selection in the design of functional parts [11].

Rafael Quelho de Macedo et al. performed an experimental and numerical analysis of the mechanical properties of ABS parts, focusing on porosity, cooling rate, and residual thermal stresses. The aim was to explain the changes in elastic modulus and strength with changing printing conditions, especially printing speeds in the range of 16–96 mm·s^−1^. The authors combined tensile tests, microscopic quantification of the pore fraction, and thermomechanical FEM (Finite Element Method) simulations in Abaqus to calculate temperature fields and residual stresses, while introducing a metric of the average cooling rate of the part. They found that higher printing speeds increase the pore fraction due to a shorter time above the glass transition temperature and thus limited diffusion/bonding of the layers, while faster cooling leads to a lower modulus of the deposited fiber, typical of amorphous polymers. They quantified that in the worst case, pores reduced the strength by approximately 9%, while residual stress contributed to a decrease of about 3.8%, highlighting the need to optimize properties by a trade-off between minimizing porosity and controlling residual stress [12].

Caterina Casavola et al. focused on quantifying residual stresses in ABS parts manufactured by FFF technology. The aim was to overcome the limitations of contact methods (e.g., strain gauges) and reliably determine the stresses arising from thermal cycling during printing. The authors combined a hole-drilling method with non-contact optical interferometry ESPI to measure the released strains and then used an orthotropic FEM model to calculate stresses. They compared four-layer deposition strategies (30°, 45°, 0°/90°, and 0°) and measured the stresses on both the top and bottom sides of the samples. They found that the 45° grid resulted in the lowest residual stress, while the 30° grid was the most unfavorable, with values up to around six MPa (more than 20% of the yield strength of ABS), with the differences between the strategies being most pronounced in the surface layer to a depth of approximately 0.2 mm [13].

Hussain Gharehbaghi et al. investigated the fatigue behavior of continuous glass fiber reinforced PLA (Polylactic Acid) honeycomb composites fabricated by FFF technology, focusing on S–N curves and especially on the development of stiffness and strength during cyclic loading. They performed tensile cyclic tests at 55%, 65%, and 75% UTS (R = 0.05) and evaluated the strength and stiffness after reaching 30%, 60%, and 90% of the average fatigue life. To eliminate delamination at the cell junctions, they used an intertwined drive path printing strategy and analyzed the failure mechanisms fractographically using SEM. They found that although continuous fibers significantly increase fatigue life, the degradation of properties is nonlinear, with stiffness decreasing faster than strength, especially in the middle phase of the life (30–60%). For example, at 60% of life and a load of 75% UTS, stiffness decreased by almost 50%, while strength decreased by approximately 20%, with matrix cracking and fiber pullout being the dominant damage mechanisms [14].

Anto Antony Samy et al. analyzed the influence of the infill pattern on the accumulation of residual stresses and warpage during FFF printing of semi-crystalline PP (polypropylene). The aim was to determine, using numerical simulation and experimental validation, which type of infill (Line 90°/90°, Line 0°/90°, Zigzag 45°/−45°, Zigzag −45°/45°, or Concentric) best minimizes thermal stress and deformations. They used a multi-physics model in COMSOL Multiphysics that linked mechanics, heat transfer, and crystallization kinetics, simulating the printing using the method of gradual activation of elements according to the nozzle path; the results were validated by 3D scanning of real samples. They found that the concentric pattern achieved the best results, reducing residual stress by 21% and strain by 5.5% compared to the reference Line 90°/90° pattern, while Zigzag 45°/−45° led to the largest increase in stress (by 31%) and Zigzag −45°/45° to the largest increase in strain (up to 37%). They attributed the differences to the different thermal histories of the individual paths, where the continuous paths of the concentric pattern promote more uniform cooling, while the short alternating paths of the zigzag modes increase local overheating and inhomogeneous solidification [15].

The aim of this article is to assess the feasibility of reflection photoelasticity (PhotoStress) for full-field, comparative visualization of near-surface residual-stress relaxation in polymer composite samples produced by material extrusion. In addition, this experiment evaluates whether an abrasive water jet (AWJ) relief cut can be used as a repeatable stress-release feature under fixed cutting conditions, while minimizing additional mechanical and thermal loading in the vicinity of the cut. A key methodological contribution of the present work is the combination of a reflection-mode PhotoStress configuration (enabled by a bonded photoelastic coating on an opaque composite substrate) with a controlled AWJ stress-relief cut to enable directly comparable full-field fringe patterns across different specimen thicknesses and between the non-annealed and annealed conditions.

## 3. Materials and Methods

This chapter describes the preparation of samples from ASA Kevlar composite filament, the selected post-processing (annealing), the method of reflective photoelasticity (PhotoStress), and the procedure for performing a release cut with an abrasive water jet, including the method of recording and evaluating the results.

### 3.1. Material and Sample Preparation

The material used was an ASA-based composite filament reinforced with aramid fibers (ASA Kevlar, Spectrum Pęcice, Poland). According to the manufacturer’s technical datasheet, the filament is intended for mechanically loaded printed parts with improved abrasion and impact resistance [16]. Key nominal material properties are summarized in Table 1.

To evaluate the influence of thickness and post-processing, samples with 100% infill of dimensions 60 × 50 mm were printed in thicknesses of 2, 4, 6, 8, and 10 mm. Each thickness level was prepared in two series: non-annealed and annealed, to compare the level of residual stress after heat treatment. For each thickness and condition (non-annealed and annealed), one specimen was manufactured (n = 1). The samples were printed on a BambuLab X1 Carbon (Shenzhen, China) using G-code generated in slicer Bambu Studio (ver. 2.3.1.51), at constant input parameters summarized in Table 2. The filament was continuously dried in a dryer at a temperature of 65 °C during printing and fed into the printer through a PTFE tube. Individual samples were printed one by one and removed only after complete cooling below 30 °C.

**Figure 1:**
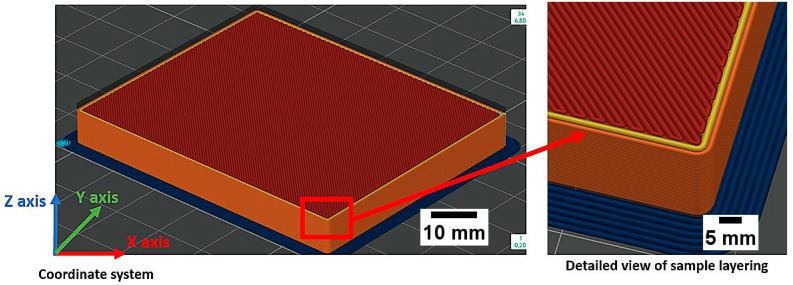
Detailed view of the sample along with the orientation of the layers.

During the production of the samples, a brim adhesion feature with a total width of 3 mm was used to improve bed adhesion and reduce the risk of warping at the corners. A detailed view of the brim and the printing orientation is shown in Figure 1.

In general, the annealing process is a heat treatment of materials to change their physical and, in some cases, chemical properties. The process involves heating the material to a certain temperature for a precisely defined time interval. In additive technologies, where polymer materials such as ABS, PC (Polycarbonate), ASA, Nylon, or materials marked as HT (Heat Treatable) are used, the annealing process can improve mechanical properties, heat resistance, and relieve residual stresses. For this reason, the prepared samples were subjected to annealing [18].

**Figure 2:**
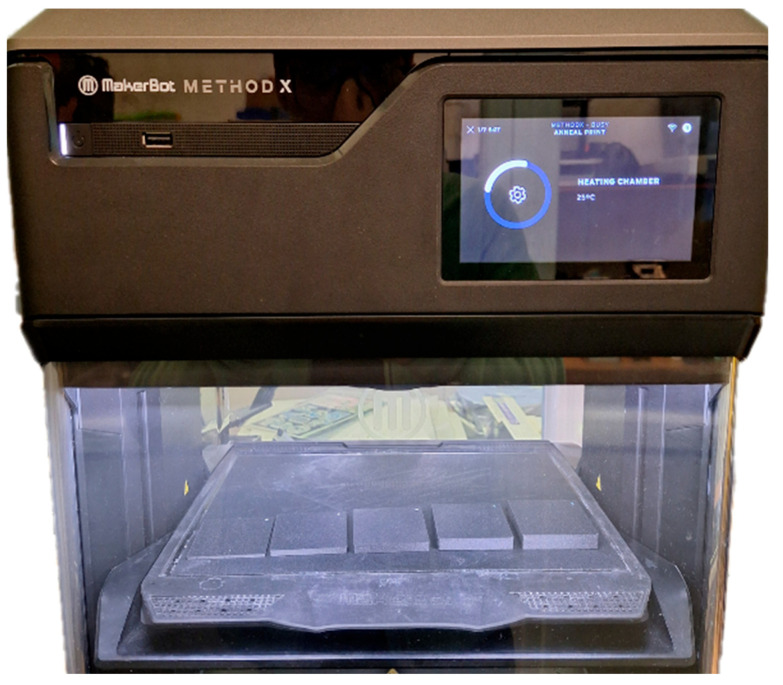
Samples in the annealing process.

The annealing temperature was chosen at 80 °C for 5 h based on the manufacturer’s stated material deformation temperature of approximately 89 °C, with the samples annealed in the Makerbot/Ultimaker Method X device, UltiMaker, Geldermalsen, The Netherlands (Figure 2) and, after the cycle, left in the device until cooled below 30 °C to minimize dimensional deformations.

Before and after annealing, the samples were weighed to determine the weight difference, which represents the removal of excess moisture. It can be seen in Table 3 that the weight difference after annealing is minimal.

### 3.2. Reflection Photoelasticity and Application of Photoelastic Coating

**Figure 3:**
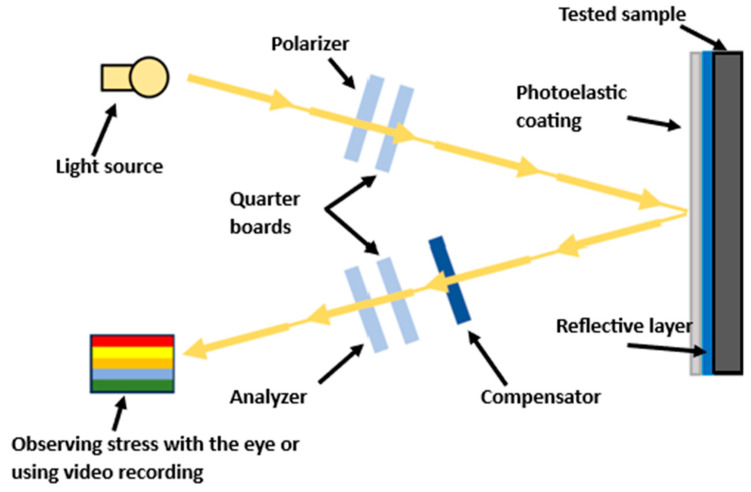
The principle of monitoring isochromatic fringes (stress) using reflective photoelasticimetry.

Photoelasticimetry is an optical experimental method for identifying residual stresses and deformations on the surface of a component. The basis is the adhesion of a photoelastic coating to the tested part; it is assumed that surface deformations are transferred to the coating. After illumination with polarized light, an isochromatic image is displayed in the polariscope, which allows the localization of the most stressed areas (see Figure 3) and their subsequent comparison using a compensator [19,20].

Residual stresses in the printed samples were visualized by the PhotoStress method based on the principle of reflection photoelasticity using a photoelastic coating. Due to the opacity of the selected composite material, a reflection configuration was used, where a fringe (isochromatic) is observed in reflection from the reflective layer of the coating.

For reflection measurements on the opaque composite specimens, a reflective photoelastic coating (Micro-Measurements PS-1) was selected because it incorporates a reflective layer suitable for reflection polariscope configurations. The coating was supplied as a flat sheet (254 × 508 × 2.05 mm). According to the manufacturer, the coating can accommodate strains up to approximately 5%, elastic modulus 2500 MPa, and has a stated maximum service temperature of 150 °C, which is compatible with the experimental conditions [21]. For the 2 mm thick specimens, given that the PhotoStress coating thickness (2.05 mm) is comparable, a coating-induced reinforcement effect can be reasonably expected. This may alter the local compliance of the specimen-coating system and reduce strain transfer into the coating, thereby decreasing the optical sensitivity of the measurement.

**Figure 4:**
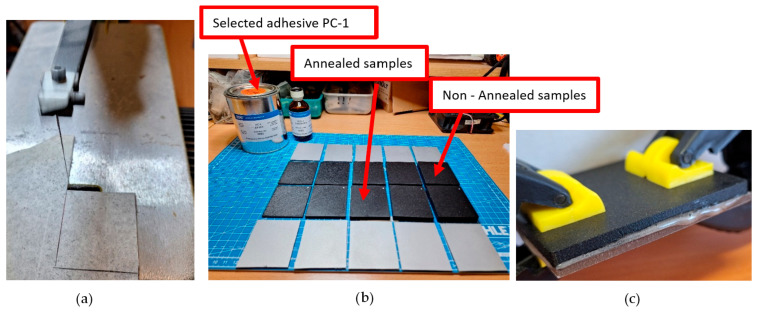
Sample preparation process. (**a**) Coating cutting process; (**b**) Adhesive used and prepared coating; (**c**) Bonding together with adhesive curing.

A two-component adhesive recommended for PS-1 coating (Micro-Measurements PC-1, Wendell, NC, USA) was used to bond the coating to the specimen surface. The adhesive was applied as a thin, uniform layer, and the coating was pressed into place to minimize air entrapment. All specimens were cured for 12 h at room temperature in accordance with the manufacturer’s instructions. The preparation process is shown in Figure 4.

**Figure 5:**
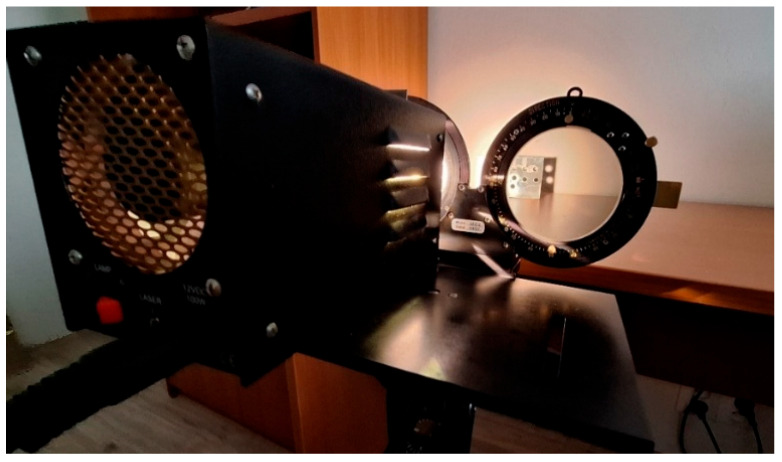
Recording stresses with the Vishay LF/Z-2 polariscope on samples.

Measurements were performed using a Vishay LF/Z-2 (Wendell, NC, USA)) reflection polariscope (Figure 5). The optical configuration (polarizer/analyzer and compensator settings) and imaging conditions were kept constant throughout all measurements to enable direct comparison between specimen thicknesses and thermal conditions. Fringe order (N) was evaluated semi-quantitatively from the recorded white-light isochromatic fringe patterns by assigning the observed fringe sequence to the standard PhotoStress fringe-order reference provided by Vishay Micro-Measurements. The fully coated front face visible within the polariscope field of view was used for specimen-wide (full-field) assessment, while the slit opening was excluded from evaluation. The global maximum fringe order (Nmax,global) was defined as the highest clearly identifiable fringe order observed within the evaluated area. For the region-based comparison, the same criterion was applied within each predefined rectangular region placed at fixed positions relative to the release cut, reporting the maximum identifiable order in that region. Because fringe discrimination in white light becomes progressively less reliable at higher orders, the reported N values are treated as comparative (semi-quantitative) indicators of changes in the isochromatic response rather than as absolute stress values.

After curing, each coated specimen was inspected in the reflection polariscope prior to cutting to establish a baseline image. Under the applied optical configuration, no discernible isochromatic fringes attributable to the cure process were observed.

### 3.3. Stress Relief with an Abrasive Water Jet

To induce a local stress-relaxation response, a straight abrasive water jet (AWJ) relief cut was introduced into each specimen. In this study, the cut acts as a controlled “stress-release feature” that locally redistributes residual stresses; the resulting near-surface strain field is then visualized using the PhotoStress method.

**Figure 6:**
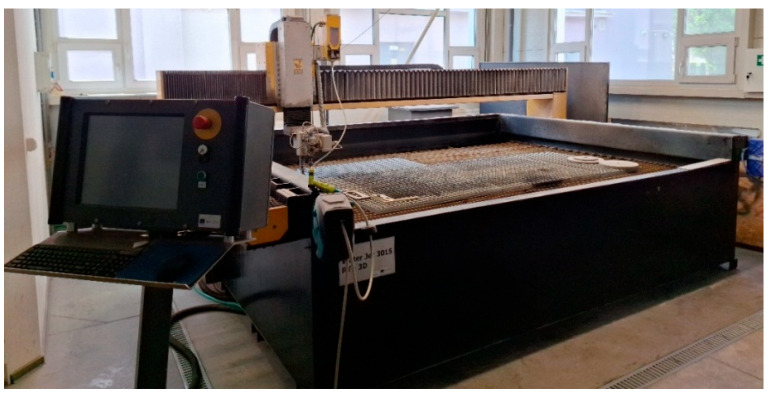
Used Water Jet Cutting Machine 3015 RT-3D.

The cutting of the samples was carried out on the Water Jet 3015 RT-3D (Kovostrojservis, Pardubice, Czech Republic) device, whose working area has dimensions of 3000 × 1500 mm (see Figure 6). The device uses a high-pressure pump type PTV 3.8/60, whose working pressure is adjustable in two levels to 50 MPa and 415 MPa. Other parameters of the machine are water nozzle diameter D0 = 0.33 mm, focusing tube diameter dv = 0.9 mm, focusing tube length dL = 76.2 mm, and cutting head inclination angle 90°. The abrasive is supplied to the cutting head using a dosing system into a container, the dosing accuracy of which is ±2 g. Australian garnet abrasive with a grain size of MESH 80 was used in the cutting process.

**Figure 7:**
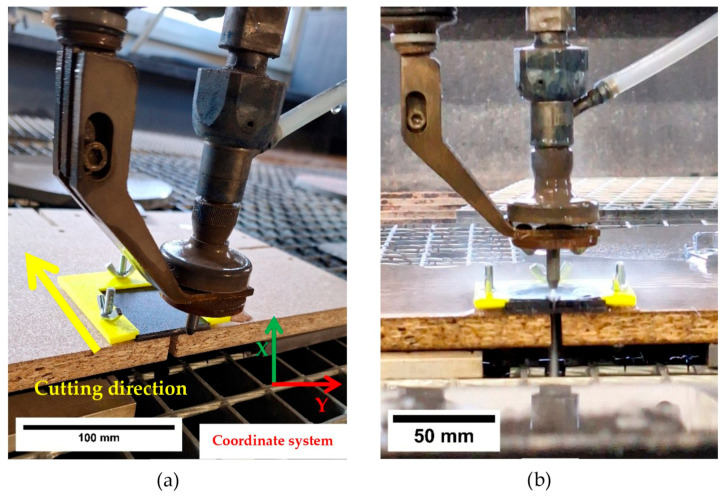
Sample cutting process. (**a**) Sample clamped in the jig; (**b**) Cutting process.

For the prepared 60 × 50 mm plates, a dedicated clamping fixture was designed to ensure repeatable sample placement and to minimize vibration and local bending during cutting (see Figure 7). The fixture constrained the specimen in all directions and included a clearance slot beneath the cutting line to prevent interaction with the cut edge and to avoid contact between the jet and the fixture.

**Figure 8:**
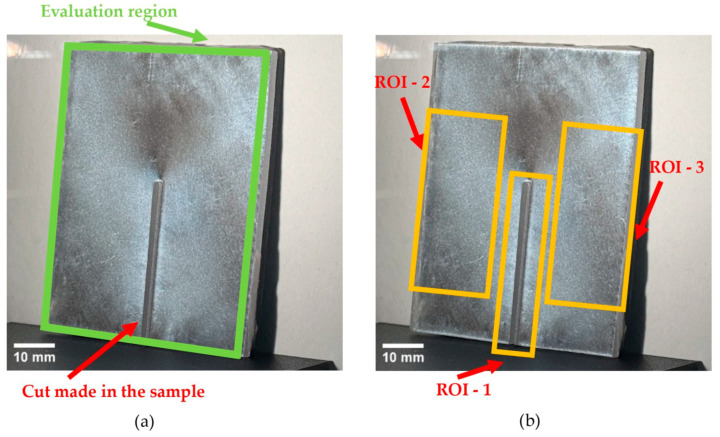
Sample with cut after recording through a polariscope: (**a**) evaluation region covering the coated front face used to determine Nmax,global; (**b**) predefined rectangular regions used for supplementary spatial comparison.

The relief cut followed a predefined path consisting of a 30 mm lead-in and a 30 mm cut length to ensure consistent cutting conditions. The parameters of the abrasive water jet (AWJ) process were kept constant throughout the experiment: stand-off distance 4 mm, water pressure 415 MPa, abrasive mass flow rate 300 g·min^−1^, and feed rate 100 mm·min^−1^. After AWJ cutting, the specimens were cleaned to remove abrasive residues and dried immediately with compressed air prior to polariscope imaging. Although water absorption in the coating/adhesive system was not quantified, the exposure time to water during AWJ cutting was short, and, during subsequent imaging under identical optical settings, no visually detectable changes attributable to moisture effects were observed (e.g., discoloration, blistering, localized optical haze, or loss of adhesion). The evaluated area was assessed by optical inspection, and no macroscopic coating or bond-related defects (e.g., local debonding, wrinkles, or visible damage) were identified. Accordingly, potential moisture-uptake effects were treated as a secondary factor and were not quantified within the scope of the present comparative experiment. A straight stress-relief cut of 30 mm (sample length 60 mm) was introduced so that the specimen remained as a single piece (see Figure 8).

## 4. Results

After the straight AWJ relief cut, the coated front face was observed in a reflection polariscope and recorded within the available field of view (Figure 8). The specimen-wide evaluation region used for subsequent full-field assessment is indicated in Figure 8a and covers the accessible coated surface, with the slit opening excluded from evaluation. Under identical optical and acquisition settings, the presence of isochromatic fringes, their spatial distribution, and their localization relative to the release cut and specimen edges were documented. The recorded fringe fields represent the optical response of the PhotoStress coating to strain redistribution associated with residual-stress relaxation triggered by local material removal (release cut). For semi-quantitative comparison, the global maximum fringe order (Nmax,global) was extracted over the evaluation region. In configurations exhibiting resolvable fringes, predefined ROIs (Figure 8b) were additionally evaluated to compare cut-adjacent and far-field responses.

**Figure 9:**
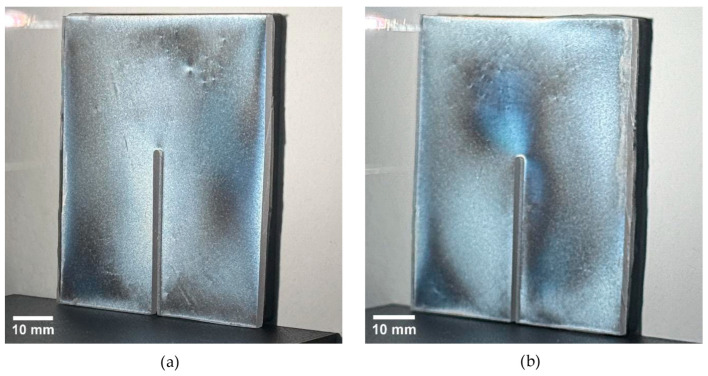
Isochromatic fringe patterns (reflection PhotoStress) on the specimen with t = 4 mm after AWJ relief cutting: (**a**) non-annealed; (**b**) annealed.

For specimens with thicknesses of 2–6 mm, no discernible isochromatic fringes were detected in either the non-annealed or annealed condition (Figure 9). Accordingly, the global maximum fringe order is reported as below the first clearly identifiable fringe-order level under the present measurement configuration (Nmax,global < 0.60; Table 4). In the PhotoStress method, the isochromatic fringe order reflects the difference between in-plane principal strains in the coating. Therefore, the absence of resolvable fringes indicates that the surface-layer principal strain difference was either low or insufficiently heterogeneous to be resolved at the sensitivity and spatial resolution of the employed optical setup.

**Figure 10:**
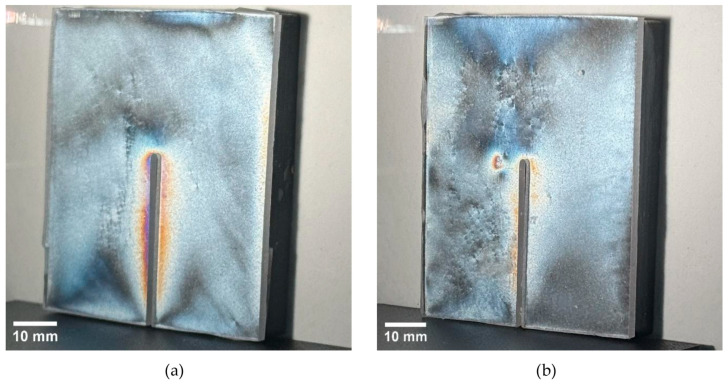
Isochromatic fringe patterns (reflection PhotoStress) on the specimen with t = 8 mm *t* = 8 mm after AWJ relief cutting: (**a**) non-annealed; (**b**) annealed.

For specimens with a thickness of 8 mm, discernible isochromatic fringes were observed primarily in the vicinity of the release cut, forming a narrow fringe adjacent to the slit and reaching the highest intensity near the rounded cut end (Figure 10). In the non-annealed condition, the global maximum observable fringe order over the specimen-wide evaluation region reached Nmax,global = 1.0 (Table 4). After annealing, the full-field response was visibly attenuated, and the global maximum decreased below the first clearly identifiable fringe-order level (Nmax,global < 0.60; Table 4). The peak response was localized in the cut-adjacent region (ROI-1), where the ROI maximum decreased from N = 1.0 to N = 0.6. In contrast, the far-field regions (ROI-2 and ROI-3) remained at N = 0.6 in both conditions. Overall, the 8 mm results indicate that annealing primarily suppresses the cut-adjacent peak response, while the far-field response remains near the first resolvable level under the present optical settings.

**Figure 11:**
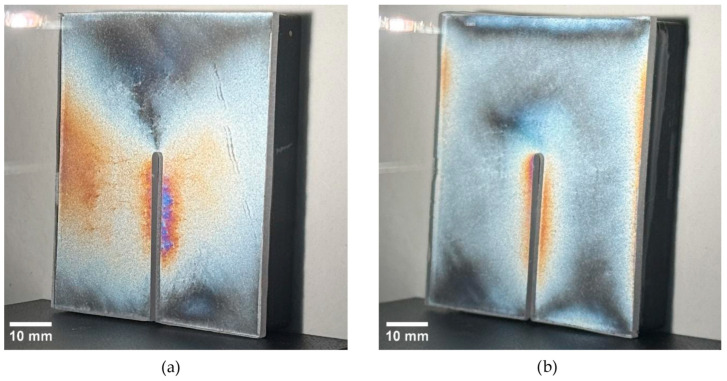
Isochromatic fringe patterns (reflection PhotoStress) on the specimen with t = 10 after AWJ relief cutting: (**a**) non-annealed; (**b**) annealed.

For specimens with a thickness of 10 mm, the isochromatic response was spatially more extensive than at lower thickness (Figure 11), with detectable low-order coloration also present in far-field areas away from the slit. In the non-annealed condition, the global maximum observable fringe order reached Nmax,global = 1.2, and was located in the cut-adjacent region (ROI-1) near the rounded termination of the release cut (Table 4). The far-field regions (ROI-2 and ROI-3) exhibited higher fringe levels (N = 0.9) than those observed after annealing (Table 5). Following annealing, the peak response remained localized in ROI-1 but decreased to Nmax,global = 0.9 (Table 4), while far-field maxima decreased to N = 0.6 in both ROI-2 and ROI-3 (Table 5). Collectively, these results demonstrate that annealing reduces both the cut-adjacent stress and the far-field fringe levels under identical optical settings.

Overall, annealing at 80 °C for 5 h reduced the full-field isochromatic response recorded after AWJ relief cutting. A semi-quantitative full-field summary based on the global maximum fringe order (Nmax,global) is provided in Table 4. No resolvable fringes were detected for thicknesses of 2–6 mm in either the non-annealed or annealed condition (Nmax,global < 0.60). In contrast, a localized peak response was observed at 8 mm in the non-annealed condition (Nmax,global = 1.0) but fell below the first clearly identifiable level after annealing (Nmax,global < 0.60). For 10 mm, a more spatially extensive response was recorded, with the global maximum decreasing from Nmax,global = 1.2 in the non-annealed condition to Nmax,global = 0.9 after annealing.

For thickness exhibiting resolvable fringes (8 and 10 mm), a ROI-based evaluation was performed to capture spatial redistribution between the cut-adjacent zone and far-field areas (Figure 8b). Table 5 summarizes the maximum identifiable fringe order (N) in each ROI before and after annealing and reports the corresponding change (Δ) between both conditions. The results show that the annealing-related reduction is concentrated in the cut-adjacent region (ROI-1). In the far-field regions, the response remains at the first resolvable level for the 8 mm specimens (N = 0.6 in both conditions), whereas for the 10 mm specimens, it decreases from N = 0.9 before annealing to N = 0.6 after annealing (ROI-2 and ROI-3).

The full-field and ROI-based records indicate that the detectability and magnitude of the PhotoStress isochromatic response after AWJ relief cutting depend on specimen thickness and thermal post-processing under the present measurement configuration. No resolvable fringes were observed for thicknesses of 2–6 mm in either condition (Table 4). At 8 mm, the response was localized near the release cut and, after annealing, decreased below the first resolvable level in the full-field evaluation (Table 4). The ROI analysis confirms that this reduction is concentrated in the cut-adjacent region (ROI-1), while the far-field regions show no measurable change (Table 5). At 10 mm, the response was more spatially extensive, and annealing reduced both the global maximum (from 1.2 to 0.9) and the far-field maxima (from 0.9 to 0.6) across ROI-2 and ROI-3. Overall, annealing at 80 °C for 5 h consistently attenuated the isochromatic fringe response in configurations where fringes were resolvable.

## 5. Discussion

The thickness-dependent fringe response observed in this study should be interpreted in the context of the present optical sensitivity and the semi-quantitative indicators adopted for comparison. The full-field global maximum fringe order (Nmax,global; Table 4) indicates that resolvable fringes were not detected for thicknesses of 2–6 mm under the applied measurement configuration, whereas distinct responses were recorded for 8 mm and 10 mm. Such thickness dependence is plausibly linked to differences in thermal history, constraint, and cooling gradients during material extrusion, which can affect the residual-stress state and its redistribution following a local stress-relief cut. Importantly, the reported differences are supported here by consistent evaluation criteria (i.e., identical optical settings and the same definition of full-field and ROI evaluation regions), rather than by qualitative image inspection alone.

Annealing at 80 °C for 5 h attenuated the isochromatic response in configurations where fringes were initially resolvable, as evidenced by both the full-field metric and the ROI-based maxima. Specifically, the global maximum decreased after annealing for 8 mm and 10 mm, and the ROI comparison indicates that the reduction is predominantly concentrated in the cut-adjacent region (ROI-1), whereas the far-field regions remain close to the first resolvable level for 8 mm and decrease for 10 mm (Table 5). This behavior is consistent with thermally activated stress relaxation in polymer-based systems, where annealing promotes molecular rearrangement and reduces internal stress gradients. Within the present dataset, the annealing effect is therefore best described as a consistent attenuation of the PhotoStress response under fixed optical and cutting conditions.

The absence of resolvable fringes in the 2–6 mm specimens must not be interpreted as the absence of residual stresses. Rather, it indicates that the near-surface optical response transferred to the bonded coating, the principal strain-difference signature responsible for isochromatics, remained below the first clearly identifiable fringe-order level under the present evaluation (Nmax,global < 0.60). Alternatively, the redistribution induced by the relief cut may have been insufficient to generate a measurable fringe field at the employed coating sensitivity and spatial resolution. Consequently, the present results are intended primarily for comparative assessment across thickness and heat-treatment conditions under identical measurement conditions.

Using AWJ to introduce a stress-relief cut is advantageous because it avoids direct tool–material contact and does not intentionally impose substantial thermal loading compared with thermally intensive cutting methods. Under constant cutting conditions, AWJ provides a controlled means of creating a localized release feature that triggers redistribution of pre-existing stresses and facilitates comparative visualization by the PhotoStress coating. Nevertheless, AWJ can locally modify the surface condition near the cut (e.g., erosion marks, micro-defects, or local roughness changes), which may influence the immediate cut-adjacent optical response. For this reason, the present interpretation emphasizes the occurrence, localization, and spatial extent of fringes and the semi-quantitative metrics extracted from standardized evaluation regions, rather than absolute stress quantification.

Overall, the present results support the feasibility of using the PhotoStress approach in combination with an AWJ relief cut as a comparative technique to reveal thickness- and heat-treatment-related differences in the near-surface optical response under fixed evaluation conditions. However, the present experiment can be interpreted on a semi-quantitative basis through the adopted full-field and ROI metrics. Nevertheless, because each condition was represented by a single specimen (n = 1) and part of the dataset lies near the detection threshold of the coating–optics configuration (Nmax,global < 0.60), the observed differences should be regarded as preliminary. Given the inherent variability of material extrusion and AWJ cutting, replication and uncertainty quantification are required to separate true process effects from specimen-to-specimen variation. Future work should therefore prioritize replication of specimens for each configuration to improve robustness against process-induced variability. Based on the present results, the analysis should be advanced primarily by calibrating the coating sensitivity for the applied coating thickness on the investigated polymer composite substrates, thereby enabling a transition from semi-quantitative comparative mapping toward quantitative stress estimation. In addition, verification of coating thickness uniformity and bond-line integrity should be performed to reduce measurement uncertainty. Addressing these limitations will enable progression from comparative visualization toward more reproducible mapping and, ultimately, quantitative assessment of near-surface residual stresses.

## 6. Conclusions

In this article, reflection photoelasticity (PhotoStress) was assessed as a full-field approach for comparative, semi-quantitative visualization of near-surface residual-stress relaxation in material-extrusion polymer composite specimens. By combining a controlled abrasive water jet (AWJ) relief cut with standardized full-field and ROI evaluation (Nmax,global and ROI maxima/Δ), the study establishes a reproducible workflow that moves beyond purely qualitative fringe inspection and enables thickness and heat-treatment related comparisons under fixed optical and cutting conditions. Within the sensitivity of the employed coating–optics configuration, no resolvable fringes were detected for thicknesses of 2–6 mm after the relief cut (Nmax,global < 0.60), whereas localized and more spatially extensive responses were recorded for 8 mm and 10 mm, respectively, and annealing at 80 °C for 5 h attenuated the fringe response where fringes were resolvable. Relative to pointwise approaches commonly used for residual-stress assessment in polymers and composites (e.g., strain-gauge hole drilling) or techniques requiring specialized instrumentation (e.g., diffraction-based methods), the proposed method offers rapid, non-contact, full-field visualization of stress-redistribution patterns and is particularly suited for comparative screening and spatial localization of stress-relaxation zones. Its principal limitations are that it provides an indirect near-surface response of a bonded coating rather than a direct through-thickness stress map, and that quantitative conversion to stress requires coating calibration and robust uncertainty control. Because each condition was represented by a single specimen (n = 1) and the coating sensitivity was not calibrated, the present findings should be interpreted as primarily comparative and semi-quantitative, supported by the adopted full-field and ROI-based mapping indicators rather than fully quantitative stress values. Future work will therefore focus on replication, coating sensitivity calibration, and more quantitative mapping of the near-surface strain/stress field, including standardized fringe-order and fringe-density metrics for benchmarking against established residual-stress measurement techniques.

## Figures and Tables

**Table 1:** Properties of the selected material [16,17].

Parameter	Value
Density	1.07 g·cm^−3^
Elongation at yield point (ISO 527 standard)	2.8%
Elongation at break (ISO 527 standard)	6%
Tensile strength (ISO 527 standard)	40 MPa
Modulus of elasticity in tension (ISO 527 standard)	2200 MPa
VICAT softening point (50 N)	94 °C
Aramid fibers reinforced	10%
Filament diameter	1.75 mm

**Table 2:** Input parameters used in the printing process.

Parameter	Value
Nozzle temperature	260 °C
Build plate temperature	90 °C
Chamber temperature during printing	42 °C
Nozzle diameter	0.4 mm
First layer height	0.2 mm
Layer height	0.2 mm
First layer speed	50 mm·s^−1^
Printing speed	50 mm·s^−1^
Infill pattern	Line infill, raster angle ±45°
Infill density	100%
Number of wall perimeters	2
Travel speed	250 mm·s^−1^
Added adhesive support–Brim width	3 mm

**Table 3:** Weight difference before and after annealing.

Sample Thickness	Weight Before Annealing	Weight After Annealing	Difference
2 mm	5.854 g	5.851 g	0.003 g
4 mm	11.695 g	11.687 g	0.008 g
6 mm	17.534 g	17.523 g	0.011 g
8 mm	23.378 g	23.367 g	0.011 g
10 mm	29.192 g	29.187 g	0.005 g

**Table 4:** Full-field semi-quantitative comparison of the PhotoStress response using the global maximum fringe order (Nmax,global) after AWJ relief cutting.

Thickness (mm)	Nmax,global (Non-Annealed)	Nmax,global (Annealed)	Full Field Interpretation
2	<0.60	<0.60	No resolvable fringes detected.
4	<0.60	<0.60	No resolvable fringes detected.
6	<0.60	<0.60	No resolvable fringes detected.
8	1	<0.60	Annealing reduces the global peak response.
10	1.2	0.9	Annealing reduces peak full-field response, response is more spatially extensive than at lower thickness.

Values < 0.60 indicate a response below the first clearly identifiable fringe order level in the employed PhotoStress evaluation.

**Table 5:** ROI-based comparison of maximum fringe order (N) between non-annealed and annealed conditions (NA/A/Δ).

Thickness (mm)	ROI-1 NA/A/Δ	ROI-2 NA/A/Δ	ROI-3 NA/A/Δ
8	1/0.6/0.4	0.6/0.6/0	0.6/0.6/0
10	1.2/0.9/0.3	0.9/0.6/0.3	0.9/0.6/0.3

NA = non-annealed; A = annealed; Δ = NA − A.

## Data Availability

The original contributions presented in this study are included in the article. Further inquiries can be directed to the corresponding author.

## Abbreviations

The following abbreviations are used in this manuscript:

FFF	Fused Filament Fabrication
ASA	Acrylonitrile Styrene Acrylate
AWJ	Abrasive Water Jet
3D	three-dimensional
CFF	Continuous Filament Fabrication
SLA	Stereolithography
PBF	Powder Bed Fusion
PEI	Polyetherimide
ABS	Acrylonitrile Butadiene Styrene
PA6	Polyamide 6
ANOVA	Analysis of Variance
FEM	Finite Element Method
PLA	Polylactic Acid
PP	Polypropylene
HT	Heat Treatable
PC	Polycarbonate
ROI	Region of Interest
